# Research on mathematics anxiety in primary school: bibliometric analysis and evaluation of trends

**DOI:** 10.3389/fpsyg.2025.1545556

**Published:** 2025-08-07

**Authors:** Hilal Uğraş

**Affiliations:** Department of Child Development, Karakoçan Vocational School, Fırat University, Elâzığ, Türkiye

**Keywords:** mathematics anxiety, primary school, bibliometric analysis, educational trends, RStudio program

## Abstract

**Introduction:**

Mathematics anxiety is a persistent barrier to effective learning, particularly in primary education. Understanding how this phenomenon has evolved in academic literature can help guide educational strategies and psychological interventions. This study aims to explore trends and key themes in research on mathematics anxiety in primary school students over a 45-year period.

**Methods:**

A bibliometric analysis was conducted using data retrieved from the Web of Science (WOS) and Scopus databases. The study included 404 articles published between 1979 and 2024. The analysis was performed using the RStudio environment with the Biblioshiny package. The research process involved study design, data collection, statistical analysis, data visualization, and interpretation.

**Results:**

Findings indicate that research on mathematics anxiety in primary school remained limited for several decades but has gained substantial attention in recent years. The field demonstrates an interdisciplinary nature, with contributions from both psychological and educational research domains. Key authors, journals, institutions, and countries contributing to the field were identified, highlighting global academic interest.

**Discussion:**

The results suggest a growing scholarly focus on the effects of mathematics anxiety, including how cognitive, emotional, and social factors contribute to its development and management. The interdisciplinary expansion reflects a deeper exploration into individual differences and their role in mathematics learning. These findings underscore the need for continued integrated research in addressing mathematics anxiety at early educational stages.

## 1 Introduction

Given the significance of mathematics in individuals’ lives, it is imperative that every student receives the necessary opportunities and support to excel in mathematics. Mathematics is an ideal field for the development of students’ intellectual competencies (Uğraş, 2019). In addition, mathematics education enables individuals to understand life and the world, generate new ideas, and develop systematic solutions to various problems (Arslan et al., 2022). In this context, individuals’ attitudes and interests towards mathematics are critical in terms of their relationship with this field. One of the important factors affecting this relationship is the emotional experiences individuals have during the mathematics learning process. Mathematics education is not only a cognitive process but also a field in which affective variables have a strong influence.

Emotional experiences directly affect students’ learning processes and academic achievement (Frenzel et al., 2007). Intensive effort and effective study habits are necessary to achieve academic success. In this process, the role of emotions in learning is critical; especially positive or negative emotions can affect students’ performance in various ways. However, the absence of anxiety or distractions during studying helps students develop the motivation to exert more effort. Emotions affect not only motivation but also cognitive functions. Emotions can positively or negatively affect student achievement by influencing long-term memory (Ashby and Isen, 1999), directing attention processes and the use of cognitive resources (Cassady and Johnson, 2002; Meinhardt and Pekrun, 2003; Papousek et al., 2012; Spielberger, 2019); triggering different forms of information processing and problem solving (Isen, 1999); and supporting self-control (Pekrun et al., 2002). Thus, students’ affective experiences have a multidimensional impact not only on learning processes but also on overall achievement in the academic context. Moreover, while a student’s cognitive competencies predict his/her success in courses, affective variables predict the student’s desire for courses and career plans in a particular field (Harackiewicz et al., 2000; Wigfield et al., 1997). In the context of mathematics, attitudes toward this field and anxiety levels have a significant impact on mathematics education and career choices (Frenzel et al., 2007; Guntur and Purnomo, 2024; Hembree, 1990; LeFevre et al., 1992; Nathan and Jackob, 2020; Quintero et al., 2022). Therefore, mathematics anxiety emerges as an important affective factor that shapes both academic achievement and professional goals for individuals. In this context, studies investigating the causes and consequences of mathematics anxiety have a large place in the literature on mathematics education (Barroso et al., 2021; Chiu and Henry, 1990; Guntur and Purnomo, 2024; Hembree, 1990; Lee, 2009; LeFevre et al., 1992; Lukowski et al., 2019; Maloney et al., 2011, 2013; Tsui and Mazzocco, 2006). Mathematics anxiety (MA) and mathematics achievement are generally negatively related (Barroso et al., 2021; Caviola et al., 2022; Commodari and La Rosa, 2021; Passolunghi et al., 2020). However, it is seen that mathematics anxiety is generally considered a unidimensional construct in the literature (Barroso et al., 2021; Hembree, 1990; Lukowski et al., 2019; Namkung et al., 2019; Quintero et al., 2022). However, this approach may fall short in understanding the multidimensional nature of math anxiety and its broad effects on individuals. For example, emotional, cognitive, and behavioral components each represent a different facet of mathematics anxiety (Gunderson et al., 2018; Ramirez et al., 2018). In this context, studies suggest that future interventions should focus on a multifaceted approach (Guntur and Purnomo, 2024; Patrick et al., 2011). Studies on whether students with high mathematics anxiety exhibit avoidance behaviors in daily learning processes, especially in the early educational stages, are limited (Quintero et al., 2022). This limitation requires a deeper understanding of the impact of mathematics anxiety on individual differences, especially at the primary school level. The primary school years are a critical period in which attitudes toward mathematics are shaped and long-term anxiety levels are determined (Barroso et al., 2021). Therefore, it is important to analyze the bibliographic data obtained from the studies conducted in this field to identify the current trends and gaps in the literature on mathematics anxiety at the primary school level. The data obtained through bibliometric analysis provide a valuable guide in terms of revealing the structural characteristics of the scientific field (Zupic and Čater, 2015) and guiding future research. In this study, by using the bibliometric analysis method, current trends, research gaps, and potential focus areas in the mathematics anxiety literature will be systematically revealed.

### 1.1 Math anxiety in primary school

Despite mathematics being a subject of great importance, many students still perceive it as difficult to understand and learn. Children, especially from an early age, perceive mathematics as an abstract concept, leading to a sense of fear of the unknown. This fear can often continue into adulthood (Lau et al., 2022; Yenilmez and Duman, 2008). Students’ anxiety about the mathematics course is evident. Many studies in the literature involving primary school children reveal that students experience mathematics anxiety (Barroso et al., 2021; Caviola et al., 2022; Commodari and La Rosa, 2021; Guntur and Purnomo, 2024; Luo et al., 2024; Luttenberger et al., 2018; Passolunghi et al., 2020; Sorvo et al., 2017; Szczygieł and Pieronkiewicz, 2022). Research shows the effect of mathematics anxiety on mathematics achievement (Israel and Olubunmi, 2014; Mutlu and Söylemez, 2018; Türk and Bedir, 2021; Yenilmez and Özbey, 2006). Math anxiety can negatively affect individuals’ academic achievement and limit their active participation in the education process. Especially for disadvantaged groups, this anxiety may indirectly make it difficult to access equal opportunities in education. This situation can be associated with the subheadings of Sustainable Development Goal 4, which aims to ensure that all students gain proficiency in mathematical skills by receiving a quality education.

Therefore, anxiety in mathematics is an important area of research. Considering that math anxiety limits individuals‘capacity to develop competence in STEM (Science, Technology, Engineering, and Mathematics) fields, developing solutions to overcome this anxiety will support SDG4’s goal of promoting lifelong learning opportunities. The increasing number of scientific studies makes it necessary to interpret and summarize the accumulated knowledge (Talan, 2021). Thus, to support uninterrupted learning and development processes, a bibliometric approach to the studies on mathematics anxiety will contribute to the identification of deficiencies and trends in the literature. This bibliometric review can provide an important basis for developing educational policies and practices to reduce mathematics anxiety. Thus, a sustainable contribution can be made in line with SDG4’s goal of improving the quality of education.

### 1.2 Bibliometric analysis

Bibliometrics is an approach that uses mathematical and statistical methods to measure and analyze scientific publications (Pritchard and Wittig, 1981). The basic data used in this method include journal titles, authors, institutions, sources, titles, terms, keywords, abstracts, subject headings, and acknowledgments (Glanzel, 2003). While bibliometric analysis reveals the intellectual structure of knowledge fields (Arora and Chakraborty, 2021), it also allows us to determine the current status of research topics, prominent hot topics, and future directions (Kapoor et al., 2018; Mishra et al., 2017; Rejeb et al., 2020, 2021). This method offers new insights to researchers, supported by the objective and quantitative nature of the methodology (Casillas and Acedo, 2007).

**Figure 1:**
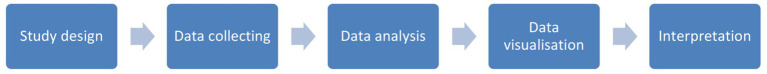
The process of science mapping.

Bibliometric analysis is also used in the application of the Knowledge Discovery from Databases (KDD) method, which allows big data to be transformed into knowledge (Lara et al., 2014). In this context, bibliometric methods can obtain meaningful information by analyzing records collected from scientific publication databases. Bibliometric analysis provides a powerful tool for the identification of research areas in different disciplines and for the quantitative and objective examination of development patterns (Song et al., 2019). Researchers often use this method to examine the scientific development of a particular topic in different fields (Dao et al., 2023). According to Zupic and Čater (2015), the process of science mapping using bibliometric approaches can be carried out systematically by following the steps shown in Figure 1. The steps include study design, data collection, analysis, visualization, and interpretation.

Study design is the stage where the purpose, scope, and methods are determined in a specific research area. Data collection involves the systematic collection of relevant scientific resources (articles, theses, etc.) from various databases. The collected data are analyzed using bibliometric methods to identify trends, important publications, and influential authors. Data visualization allows the results of the analysis to be presented through graphs and maps, thus making the data more understandable. Lastly, the interpretation stage involves evaluating the significance of the findings and the advancements in the research field. These steps help conduct the research in a systematic way and better understand the results obtained.

## 2 Method

The main aim of this research is to examine the development of research on the mathematics anxiety of primary school students over time with a bibliometric approach. The studies published in the Web of Science (WOS) and SCOPUS databases between 1979 and 2024 were analyzed from a bibliometric perspective. Thus, by determining the progress and trends of research in this field, it is aimed to contribute to the existing body of knowledge and to provide a framework for preventing repetitive studies. Reviews in the literature typically use only information sources based on a single database. For example, in bibliometric analyses, only the Scopus (Cao, 2024; Donthu et al., 2021; Rejeb et al., 2022; Wahyuni et al., 2025) database or WOS (Cao, 2024; Ersozlu and Karakus, 2019; Fu et al., 2024; Sagarduy et al., 2024) database was used. This situation causes data loss and limits the holistic view of the literature. In this study, all the research in both databases (SCOPUS and WOS), which contain the leading research, was examined by combining them, and thus it was aimed to provide a new and inclusive perspective to the literature.

Scopus has one of the most extensive data pools in terms of content coverage and is therefore used as a primary source in many bibliometric studies (Parlina et al., 2020). WOS, on the other hand, systematically indexes the scientific literature and provides high reliability in citation relationships and publication network analyses (Cobo et al., 2015). The fact that some journals indexed by Scopus and WOS are not included in other databases highlights the scope of these databases (Gavel and Iselid, 2008; Valente et al., 2022). The advanced query engines of Scopus and WOS and their high compatibility with bibliometric software (e.g., VOSviewer, CiteSpace, Bibliometrix) provide users with significant advantages in terms of visualization and analysis (Archambault et al., 2009). In addition to Scopus and WOS, some databases have extensive data networks but are not suitable for bibliometric analysis. For instance, Google Scholar offers extensive access to the literature, but for a various of reasons, it is not suitable for systematic bibliometric analysis. The metadata in Google Scholar is generally of low quality, DOI information is often missing, and the interface provided for exporting data is limited (Delgado López-Cózar et al., 2019; Martín-Martín et al., 2021). Additionally, issues such as the lack of transparency in indexing criteria, inconsistent coverage of non-English literature, and the indexing of non-existent journals reduce data reliability (Clermont and Dyckhoff, 2012; Wouters and Costas, 2012). There are even findings that the Google Scholar citation system is susceptible to manipulation (Delgado López-Cózar et al., 2019). Although the Google Scholar database contributes to the literature, it does not sufficiently support the comprehensive citation relationships, author collaborations, and data transfer tools required for bibliometric analysis. Archambault et al. (2009) found a high correlation between the number of articles calculated using Scopus and WoS and the number of citations received per country, concluding that both databases are suitable tools for scientific measurement analysis. Despite the databases’ breadth, it was found that some journals are only in WoS, some are in both WoS and Scopus, and some are only in Scopus. Therefore, the study combined the Scopus and WOS databases. Therefore, the study combined the Scopus and WOS databases.

In this context, the aim of this study is to perform a bibliometric analysis of the studies on mathematics anxiety in primary school. This study aims to provide answers to the following research questions:

- *Which are the most cited journals, the most influential authors, and the most productive institutions where studies on mathematics anxiety in primary school are published*?
- *What are the dominant themes that stand out in the studies on mathematics anxiety in primary school based on title, abstract, and keyword analyses, and how have these themes changed over the years*?

### 2.1 Data analyses

Within the scope of this research, studies on mathematics anxiety in primary school between 1979 and 2024 (05.10.2024) were analyzed. No filtering was made regarding the language of the publications. The sample consists of early access articles published in journals, as they are assumed to be the repository of the most recent findings and empirical results (Dinić and Jevremov, 2021). There are several reasons for excluding other types of publications. For example, editorial materials represent a rather heterogeneous category, and their frequency of publication within a year is quite low, which makes it difficult to calculate the relevant bibliometric statistics (van Leeuwen et al., 2013). The scientific impact of meeting abstracts and conference proceedings is quite limited; also, unlike articles, they usually do not contain reference lists, thus making citation-based analysis difficult. Book chapters and reviews, on the other hand, often provide a general summary of previous findings but may ignore current trends (Radević and Milovanović, 2024).

The reviewed studies were selected on specific topics. Words were carefully selected using predefined search terms and criteria to minimize selection bias and capture as many relevant studies as possible (Ersozlu and Karakus, 2019; Sagarduy et al., 2024). The search was conducted using the keywords (‘mathematical anxiety’ OR ‘math anxiety’) AND (‘primary school’ OR ‘elementary’) as the subject title. In the research, using R program and Biblioshiny software tools, the RStudio program was used to prevent the repetition of the articles scanned jointly in the two databases (Aria and Cuccurullo, 2017). Analyses including annual publication analysis, word analysis, analysis of journals, analysis of institutions, citation analysis, bibliographic links, co-authors, word analysis, thematic evolution of keywords, and trend issues were carried out to answer the research questions.

**Figure 2:**
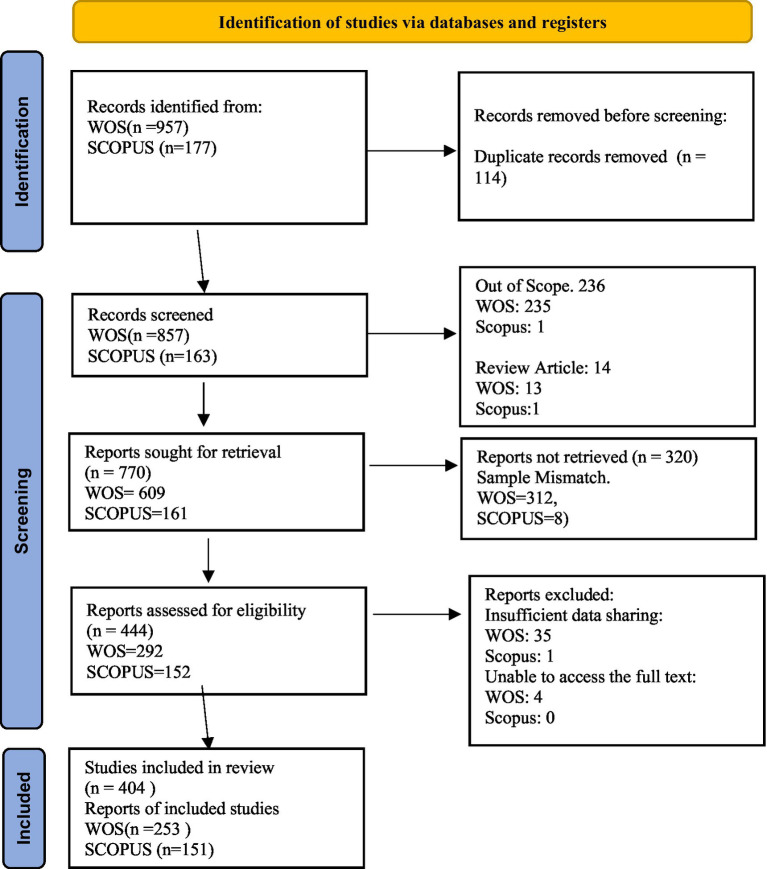
PRISMA review process.

The initial search results in the databases yielded a total of 1,134 articles, as shown in Figure 2. After reviewing the articles based on criteria such as publication date, title review, scope, and sample compatibility, 690 articles were excluded. The remaining 444 articles were examined in greater detail. Forty articles were excluded due to insufficient data sharing and lack of access to full text. The remaining 404 articles were carefully and comprehensively reviewed in terms of their relevance to our criteria and research questions. A thematic analysis procedure was followed during this process (Braun and Clarke, 2006). The researcher read each article several times to accurately understand its scope, methods, and findings. Insufficient sharing of empirical data and inability to access full texts led to the exclusion of forty articles. The remaining 404 articles that comprised the dataset were included in the bibliometric analysis. Figure 2 presents the PRISMA process.

Within the scope of bibliometric methodology, both performance analysis and scientific mapping techniques were used. For performance analysis, the number of publications and citations was determined as the basic parameters. In scientific mapping, the co-occurrence analysis method was applied (Donthu et al., 2021). The most frequently used keywords, citation analyses, and co-citation analyses; overlay, cluster density, and network maps were visualized (van Eck and Waltman, 2022).

### 2.2 Findings

The bibliometric analysis in this chapter includes the following five components; (1) some descriptive statistics on the dataset, such as the number of publications over time; (2) citation analyses, including citation patterns, including the most cited publications and authors; (3) authorship analysis, with an author productivity report and authorship indicators; (4) performance analysis of institutions, considering productivity in terms of publications and citations; (5) keyword analysis, tracking the most frequently used keywords and trends in the use of these words.

The reporting of documents on mathematics anxiety at the primary school level began in 1979. Table 1 summarizes the descriptive statistics of this bibliometric dataset.

**Table 1:** Main information about the data field of mathematics anxiety in primary school.

Description	Results
Timespan	1979-September 2024
Sources (Journals)	211
Documents	404
Keywords Plus (ID)	653
Author’s Keywords (DE)	863
Authors	1,101
Authors of single-authored docs	42
Single-authored docs	47
Co-Authors per Doc	3,49
International co-authorships %	19,8

211 journals published 404 articles between 1979 and 2024. In the field of mathematics anxiety at the primary school level, 1,101 researchers worked; 47 of the researchers worked with a single author, and 653 keywords emerged in the published studies.

### 2.3 Analysis of annual publications

**Figure 3:**
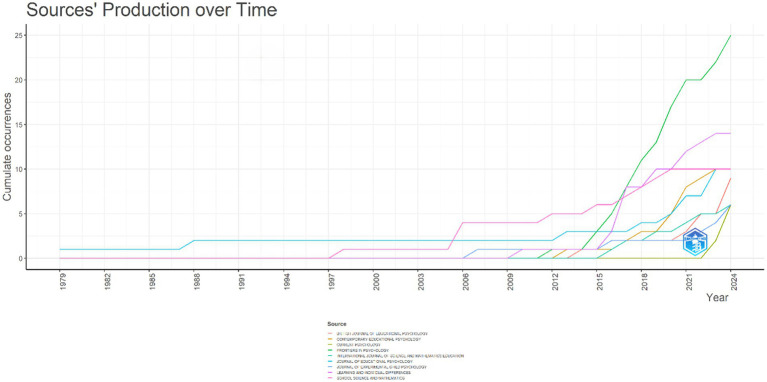
Annual research publications field of mathematics anxiety in primary school.

Figure 3 presents an analysis of the annual publications on mathematics anxiety in primary school. This analysis reveals a significant increase in researchers’ interest in the topic. Starting in 1979 and extending to 2024, the data show the evolution of academic interest in mathematics anxiety from a longitudinal perspective.

Between 1979 and 2009, a maximum of 7 articles were published annually. From 2010 to 2016, there was a consistent annual increase in the number of articles published. As of 2017, the number of articles published per year started to increase significantly; the number of 34 articles published in 2017 reached 96 before the year 2024 was completed. It is seen that there has been a break in the number of publications on mathematics anxiety in primary school, especially since 2013, and after this date, the number of publications has increased significantly, and the number of research on the subject has gained momentum over the years. The journal with the highest number of publications on mathematics anxiety in primary school was *Frontiers in Psychology* (25 articles), followed by *Learning and Individual Differences* (14 articles) and *Contemporary Educational Psychology* (10 articles). Consequently, the period from 1979 to 2024 (September) saw the publication of 404 articles.

### 2.4 Word analysis

**Figure 4:**
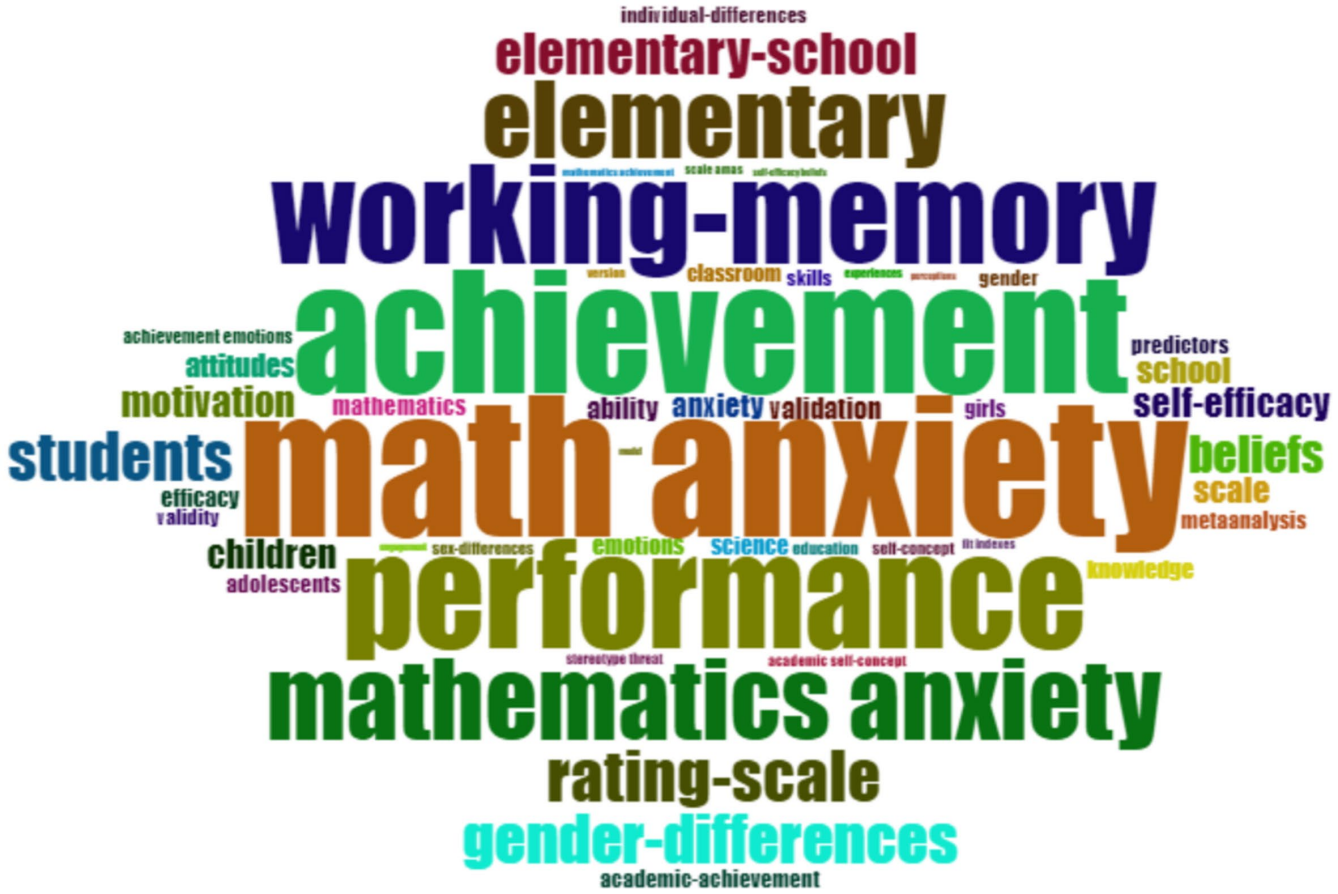
Word cloud based on keywords related on field of mathematics anxiety in primary school.

Keyword analysis was conducted to reveal important concepts related to mathematics anxiety in primary school. The frequency of keywords was visualized in the word cloud (Figure 4) related to mathematics anxiety in primary school. Such visualizations help you quickly identify prominent themes and concepts in a research area. The analysis of keywords and their frequencies provides valuable insights into current research trends priorities and areas of interest in the field.

As a result of the analysis, the term ‘math anxiety’ stands out in the word cloud with the highest frequency with 159 repetitions. In addition to this term, the words ‘achievement’ (138 repetitions) and ‘performance’ (123 repetitions) also reached remarkable frequencies. These results reveal that the terms ‘achievement’ and ‘performance’ have a significant effect on students’ mathematics anxiety. These findings are important for the development of educational practices and strategies.

**Figure 5:**
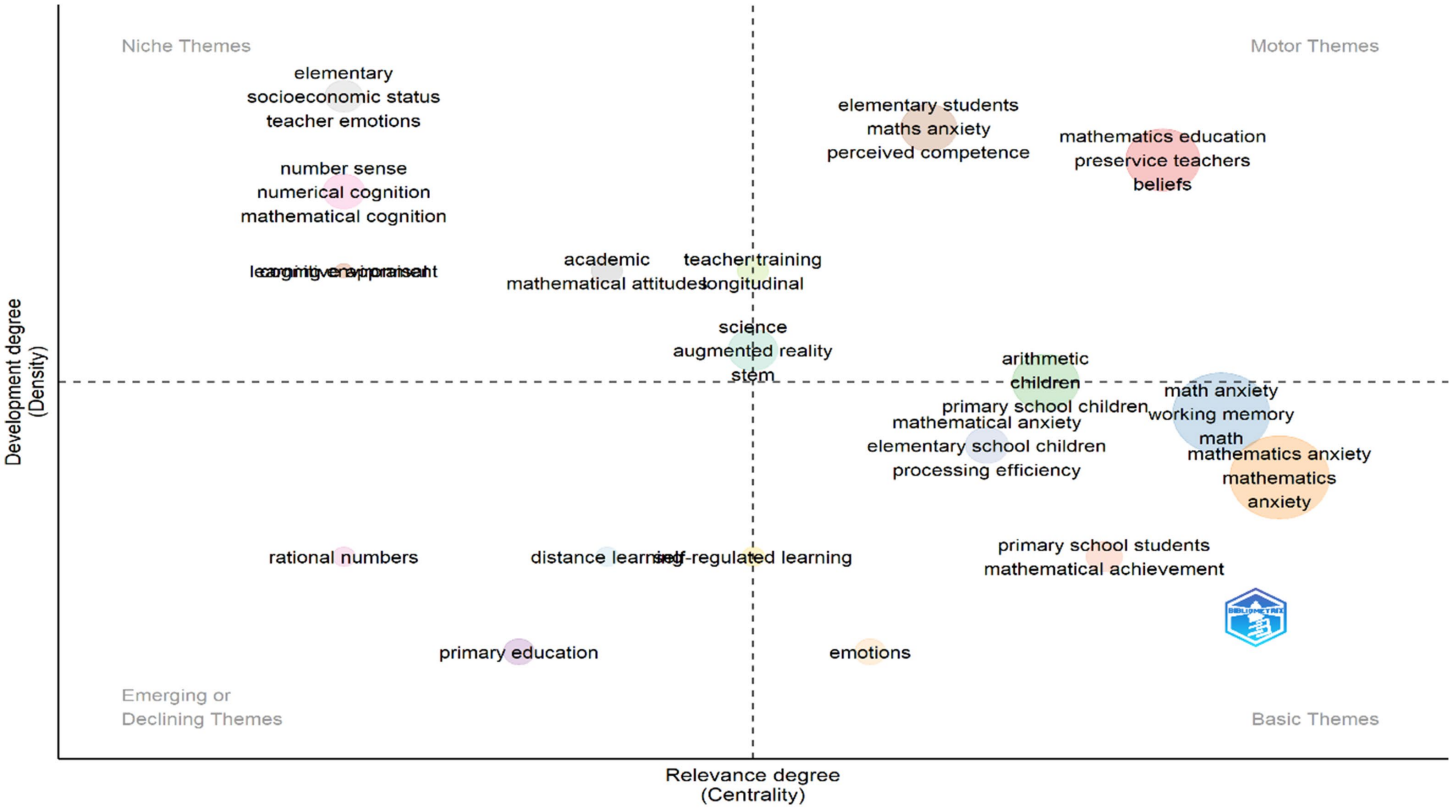
Thematic map of keywords in research on field of math anxiety in primary.

The thematic map (Figure 5) was created to visually show the research environment related to mathematics anxiety in primary schools, highlighting the main themes, specific areas, emerging trends, and key issues based on their importance and development.

According to the thematic map in the figure, the main themes of ‘arithmetic children,’ ‘mathematical anxiety,’ ‘working memory,’ ‘math anxiety,’ ‘elementary school children,’ ‘mathematics,’ ‘mathematics anxiety,’ ‘primary school students,’ ‘emotions,’ and ‘mathematical achievement’ are in the first quadrant. These themes provide well-established and in-depth connections within the framework of the current research, indicating that they are common focal points in the literature. Their positioning suggests that they are established research areas with a significant influence on other topics within the field (e.g., Munir et al., 2022).

In the second quadrant, the special themes are ‘mathematics education,’ ‘preservice teachers,’ ‘elementary students,’ and ‘maths anxiety.’ These topics are highly developed but more isolated, indicating that they are specialized within the field. Their high degree of development indicates a depth of knowledge in these areas, while their low centrality indicates that they have limited links to the wider research environment and are addressed in specialized journals or in different research communities (e.g., Kokol et al., 2022).

The third quadrant includes the themes ‘elementary,’ ‘socioeconomic status,’ ‘teacher emotions,’ ‘number sense,’ ‘numerical cognition,’ ‘mathematical cognition,’ ‘teacher training,’ ‘academic mathematical attitudes,’ ‘science,’ ‘augmented reality,’ ‘learning environment,’ ‘STEM,’ and ‘longitudinal design.’ Their position suggests that they are gaining or losing relevance but have not yet established strong links with other research areas or reached a high level of development within the field (e.g., Odden et al., 2020). In the fourth quadrant, ‘rational numbers,’ ‘primary education,’ ‘distance learning,’ and ‘self-regulated learning’ are among the key themes. These are core topics that are less developed yet have a high potential for future growth. They are important for the structure of the research field and represent emerging areas that have the potential to develop into future highlights or open up new directions in research (e.g., Liu and Yu, 2023).

### 2.5 Analysis of journals

The distribution of mathematics anxiety research in primary school across various journals provides important information about the academic settings most receptive to this interdisciplinary area of study.

**Figure 6:**
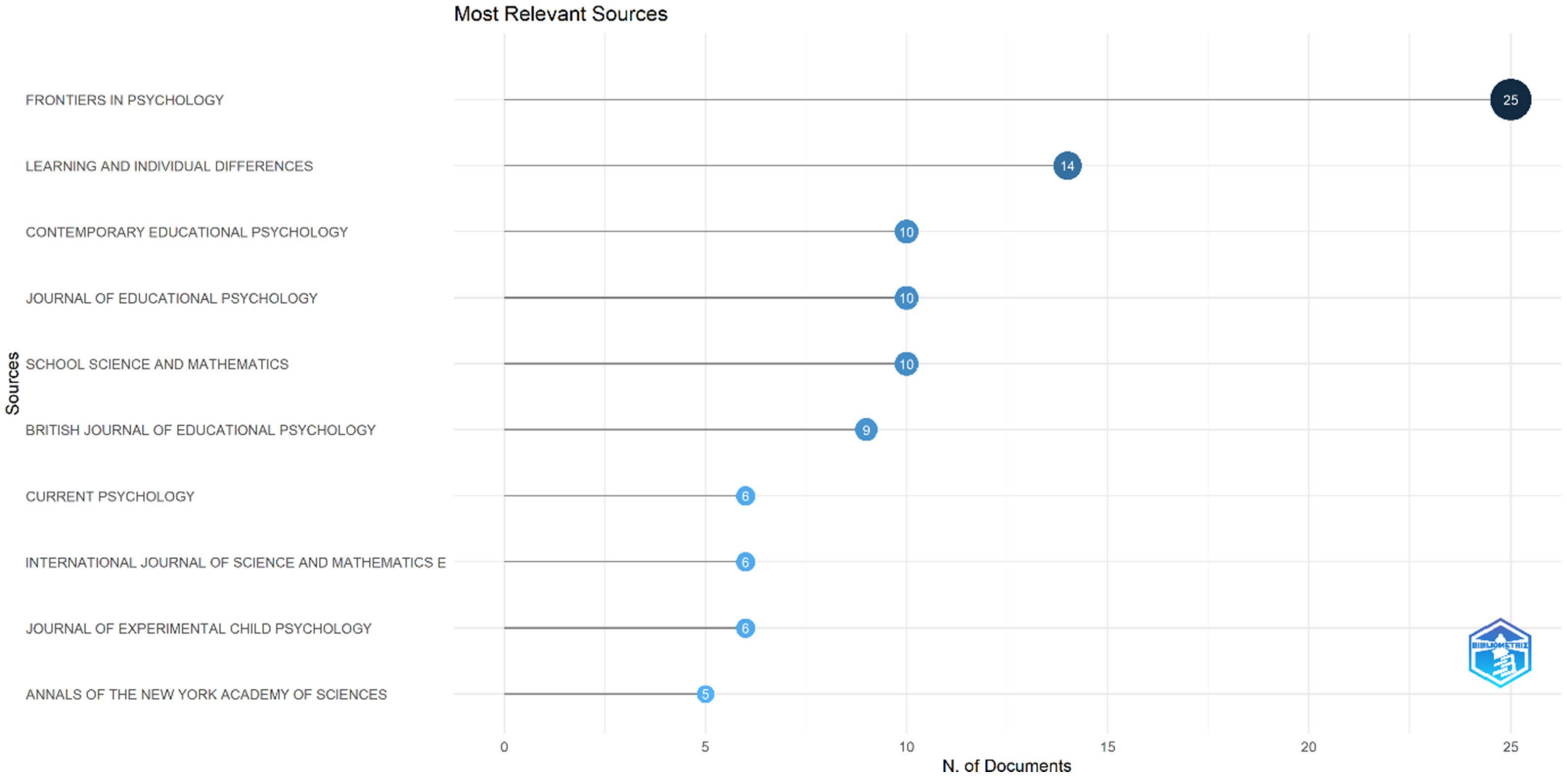
Most relevant sources on field of mathematic anxiety on primary.

Among these journals, Frontiers in Psychology (25 articles), Learning and Individual Differences (14 articles), Contemporary Educational Psychology, Journal of Educational Psychology, and School Science and Mathematics (10 articles each) stand out (Figure 6). In addition, there has been a significant increase in the number of publications on mathematics anxiety in primary schools in these journals since 2017 (Figure 6). The most cited journals were Journal for Research in Mathematics Education (486 citations), Psychological and Cognitive Sciences (483 citations), and European Journal of Psychology of Education (350 citations).

### 2.6 Cooperation between institutions and countries

In this section, the institutions that contributed the most to the research on mathematics anxiety in primary school and the centers of inter-institutional collaboration were identified. This analysis reveals the global distribution and impact area of the research.

**Figure 7:**
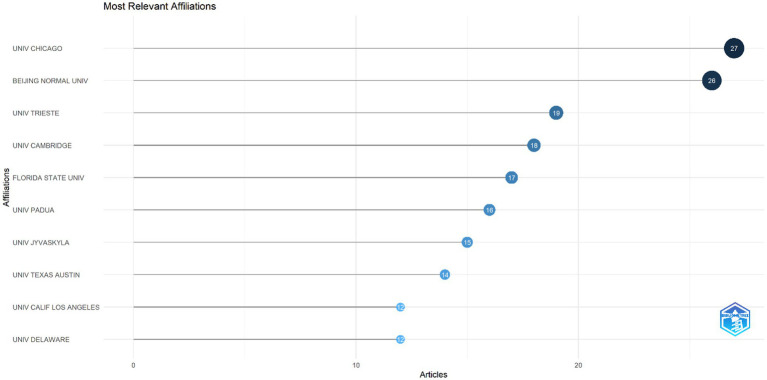
Top 10 productive institutions in the field of mathematic anxiety on primary.

Figure 7 shows the institutions with the highest number of publications. The institutions publishing on mathematics anxiety in primary school are as follows: University of Chicago (27 articles), Beijing Normal University (26 articles), University of Trieste (19 articles).

**Figure 8:**
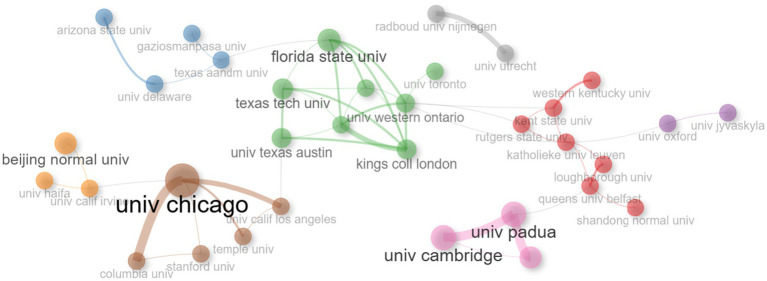
The most revelant countries field of mathematics anxiety in primary education.

An analysis of the cooperation network between these institutions revealed eight clusters. Figure 8 reveals the University of Chicago’s central location and high level of collaboration. In particular, the intensive collaboration with Stanford University, Columbia University, and Temple University reveals that this university leads research in the field of mathematics anxiety. In a different cluster, intensive cooperation between Unv. Tesas Austin, Texas Tech University, the University of Western Ontario, Florida State University, and King’s College London is noteworthy. In addition, it is seen that the University of Cambridge, the University of Trieste, and the University of Padua work in cooperation both within themselves and with universities in other clusters through the University of Padua.

**Figure 9:**
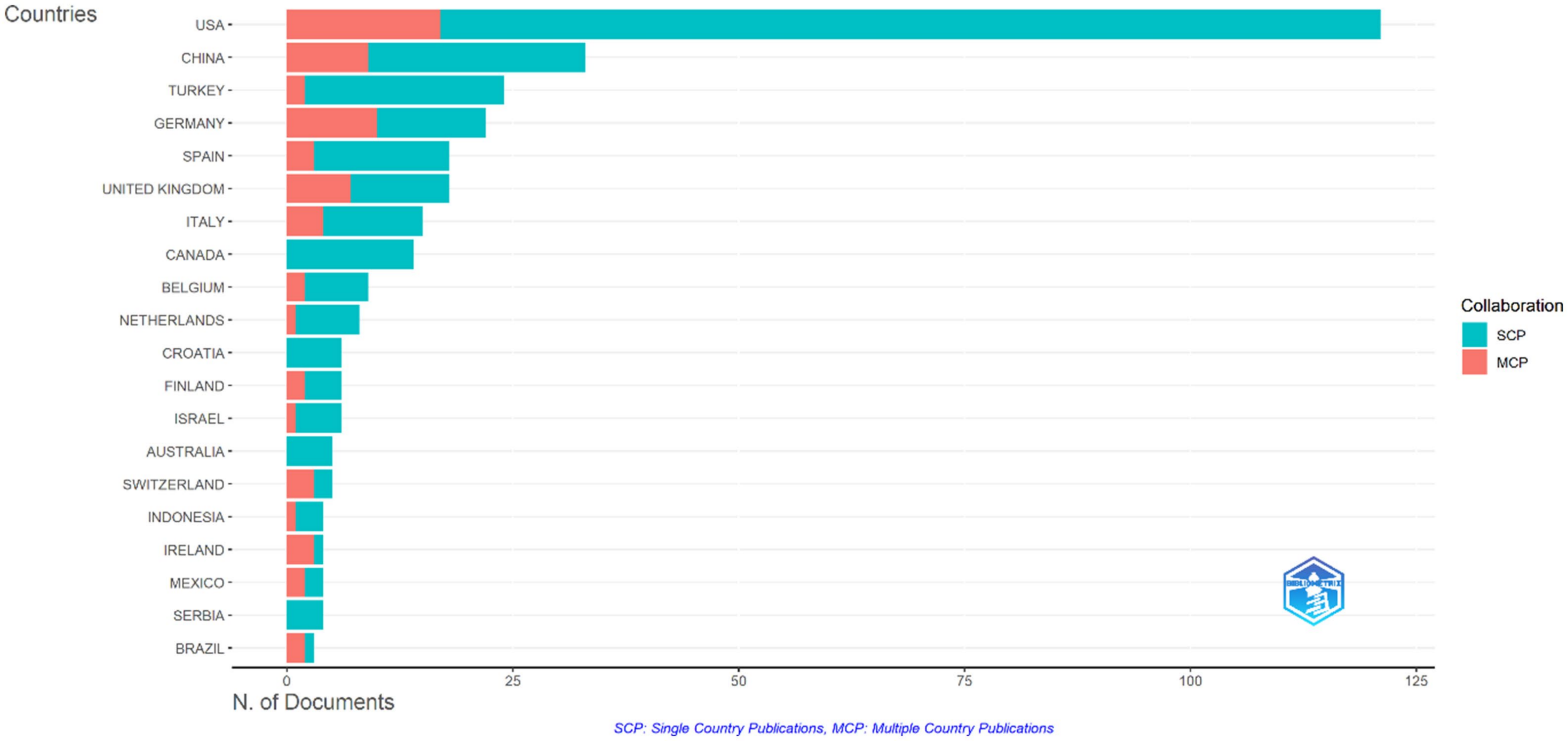
Network for inter-institutional collaboration in the field of mathematics anxiety in primary education.

The USA has the highest number of publications (121), accounting for 30% of all publications in the area of mathematics anxiety in primary school (Table 2 and Figure 9). The SCP (86%) was found to be more oriented towards national studies, and the MCP (14%) was determined to be less oriented towards international collaboration. China has 33 publications, accounting for 8.2% of the total number of publications. China makes a significant contribution to international cooperation with an MCP rate of 27.3%. The rate of SCP (72.7%) is slightly lower compared to the USA. Türkiye’ s share is 5.9% with a total of 24 publications, 91.7% of which were produced at the national level (SCP), and only 8.3% were produced in international collaboration. This evidence shows that Türkiye focuses more on national studies. Although Germany ranks 4th with a total of 22 publications, it is in a leading position in international collaboration with 45.5% MCP. Spain conducted 83.3% of its publications at the national level and 16.7% in international cooperation. This data shows that Spain has a higher MCP rate than the USA and Türkiye.

**Table 2:** Publication performance and collaboration distribution of countries in research on mathematics anxiety in primary school.

Country	Articles	Articles %	SCP	MCP	MCP %
USA	121	30	104	17	14
China	33	8.2	24	9	27.3
Türkiye	24	5.9	22	2	8.3
Germany	22	5.4	12	10	45.5
Spain	18	4.5	15	3	16.7

**Figure 10:**
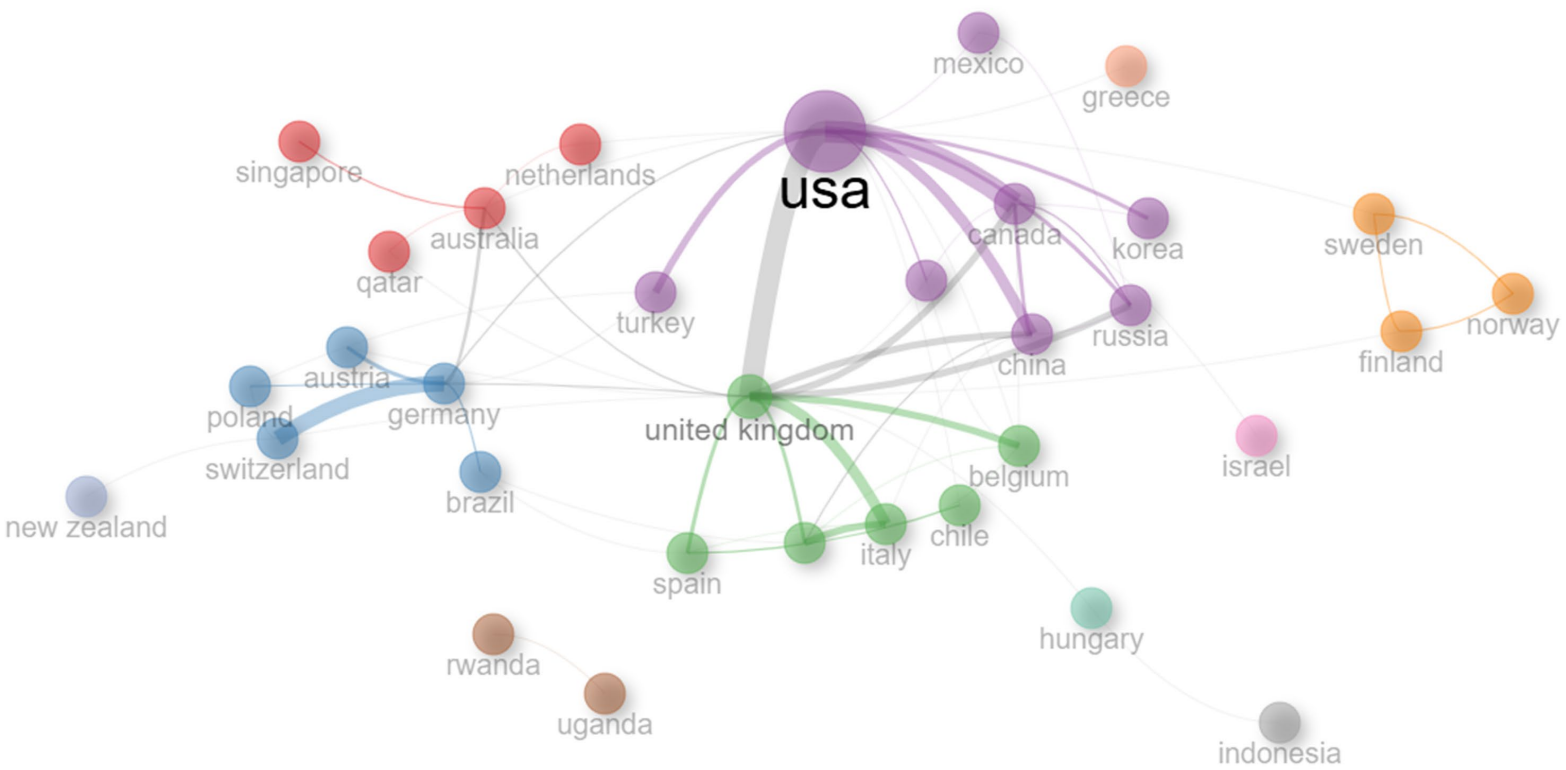
Inter-country co-operation field of mathematics anxiety in primary school.

When the cooperation network between countries in Figure 10 is analyzed, it is seen that 11 clusters are formed. Each cluster symbolizes sub-groups depicted by varying colors, exhibiting intense cooperation. The United Kingdom and the USA stand out as the most central nodes of the clusters, and these countries serve as an important bridge between other clusters. These countries are some of the most influential nodes across the network and play a critical role as information and cooperation centers. Countries in other clusters were found to be in a more isolated position as they had fewer connections or only limited cooperation within their cluster.

## 3 Attribution analysis

### 3.1 Analysis of top-cited documents

**Figure 11:**
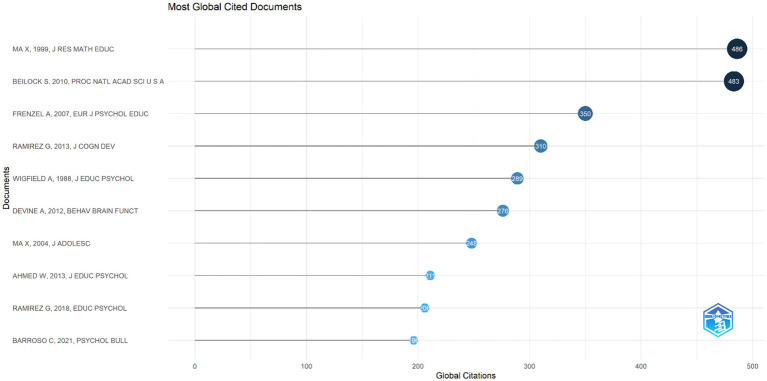
Top 10 most globally cited documents field of mathematics anxiety in primary school.

The analysis of the most cited articles in the field of mathematics anxiety in primary school (Figure 11) provides a window into the most influential research themes and methodologies shaping the current state of the field. By helping to identify important concepts and methodologies in the field, this analysis reveals key reference points in mathematics anxiety and provides critical information that can inform future research.

The most cited articles between 1997 and 2024 are shown in Figure 11. The article ‘*A Meta-Analysis of the Relationship between Anxiety towards Mathematics and Achievement in Mathematics*’ published by MA (1999) in the Journal for Research in Mathematics Education is considered one of the most cited pioneering articles in the field of mathematics anxiety in primary school with 486 citations. The article ‘*Female teachers’ math anxiety affects girls‘math achievement*’ published by Beilock et al. (2010) in Psychological and Cognitive Sciences ranks second with 483 citations. Also, Frenzel et al. (2007) article ‘*Girls and Mathematics - A “hopeless” issue? A control-value approach to gender differences in emotions towards mathematics*’ is among the three most cited studies, with 350 citations.

### 3.2 Analysis of authors

Analysis reveals that the most prolific authors conducting studies on mathematics anxiety at the primary school level have a range of publication outputs over the observed period, from six to twelve papers per author. Table 3 presents the 10 most relevant authors in the field of mathematics anxiety in primary school.

**Table 3:** Top 10 most relevant authors field of mathematics anxiety in primary school.

Author	Number of documents	Affiliacitions	Country	Total citations
Beilock S	12	Unıversıty Of Chicago	USA	1,582
Levine S	10	Unıversıty Of Chicago	USA	1,199
Passolunghi M	9	University of Trieste	Italy	294
Wang Z	9	The Ohio State University	USA	321
Ramirez G	8	Unıversıty Of Chicago	USA	1,383
Szucs D	8	Unıversıty Of Cambridge	United Kingdom	791
Mammarella I	7	University of Padova, Padova	Italy	298
Devine A	6	Unıversıty Of Cambridge	United Kingdom	812
Maloney E	6	Unıversıty Of Chicago	USA	631
Pellizzoni S	6	University of Trieste	Italy	70

Leading authors include Beilock, S. (12 papers, University of Chicago, USA), Levine, S. (10 papers, University of Chicago, USA), Passolunghi, M. (9 papers, University of Trieste, Italy), Szucs, D. (8 papers, University of Cambridge, UK), and Ramirez, G. (8 papers, University of Chicago, USA). The number of publications of the authors with the highest number of publications, especially in 2018, is also shown in Table 3. Among these authors, Beilock, S. (1,582 citations) and Ramirez, G. (University of Chicago, USA) are the most cited authors with 1,383 citations, followed by Levine, S. (1,199 citations).

Table 4 lists the most relevant countries for mathematics anxiety in primary school. When citation performance by country is analyzed, the USA (5,533 citations, 45.70%) has the highest impact in this field. The USA is followed by the United Kingdom (1,048 citations), Germany (836 citations), Canada (645 citations), China (413 citations), and Türkiye (390 citations).

**Table 4:** The most relevant country field of mathematics anxiety in primary school.

Country	Total citations	Average article citations
USA	5,533	45,70
United Kingdom	1,048	58,20
Germany	836	38,00
Canada	645	46,10
China	413	12,50
Türkiye	390	16,20

**Figure 12:**
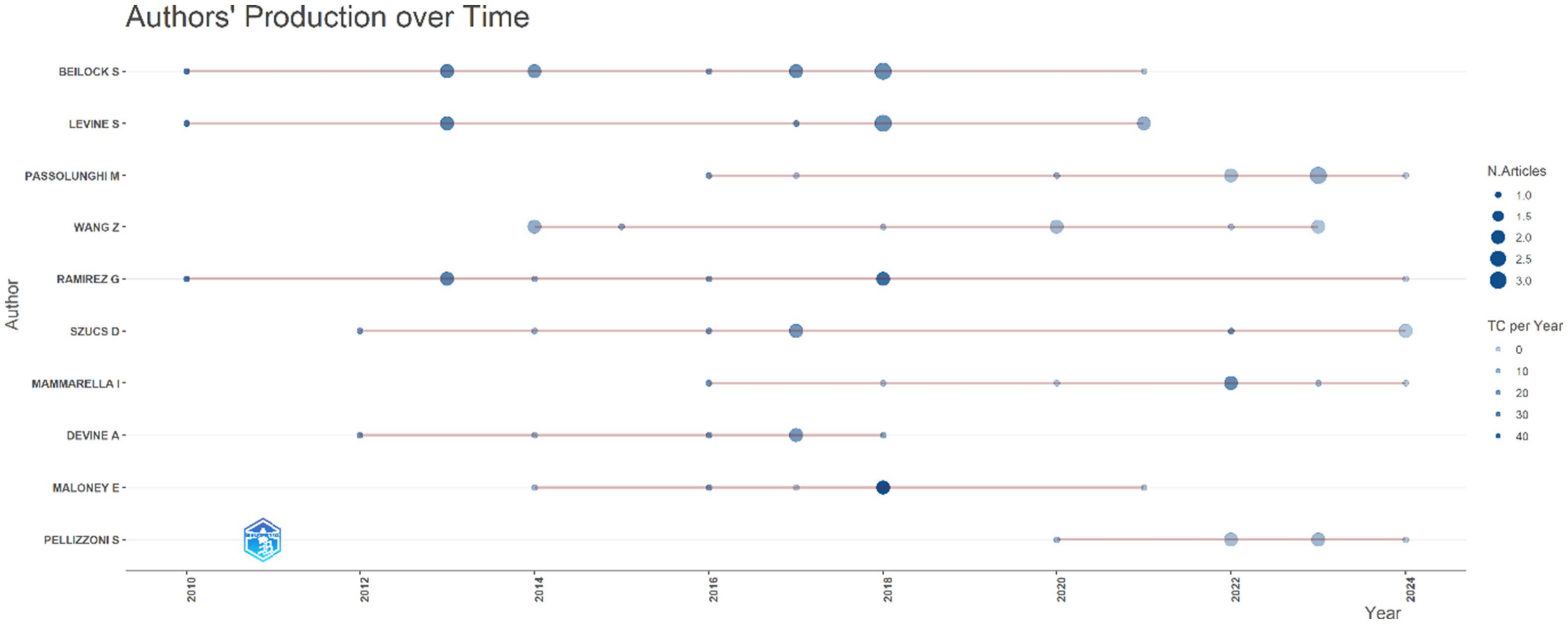
Top 10 authors’ production over time field of mathematics anxiety in primary school.

It is seen in Figure 12 that the number of publications by these authors has increased since 2010; in 2018, there were intensive publications on mathematics anxiety in primary school.

**Figure 13:**
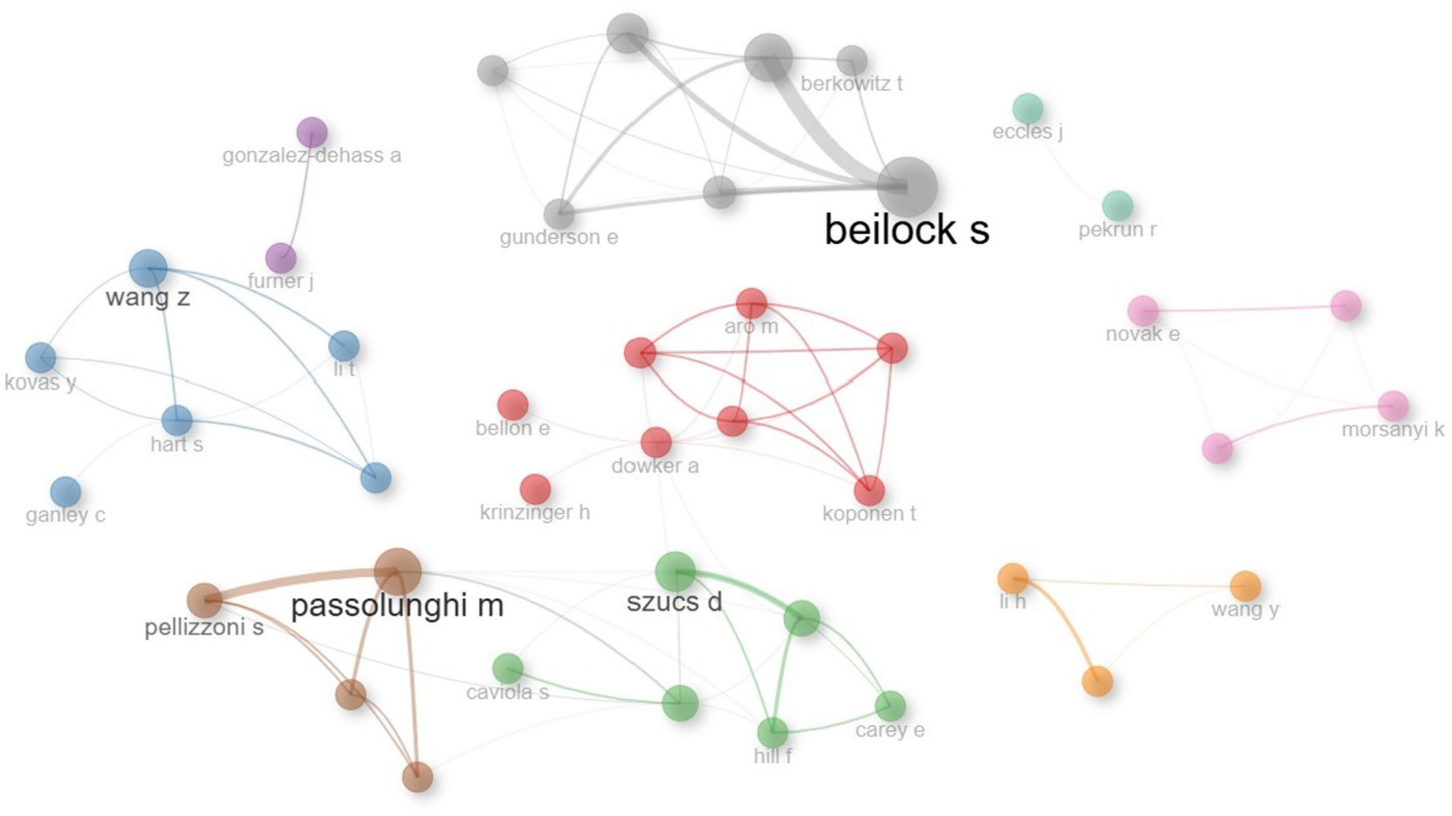
Authors’ collaboration network of the 40 most productive authors of field mathematics anxiety in primary school.

In the visual information map of co-authors in Figure 13, the size of the author nodes is shown in proportion to their publication output, and the connecting lines represent the collaborations between authors. In this context, the most central and influential nodes of the network are authors such as Dowker, A., Beilock, S., Szucs, D., and Passolunghi, M. in their research on mathematics anxiety in primary school. These authors are the key figures that direct information flow and collaboration by being at the critical points of the collaboration network. Although they have cooperation in other clusters, they are effective within their own cluster.

### 3.3 Analysis of trends topics and thematic evolution

**Figure 14:**
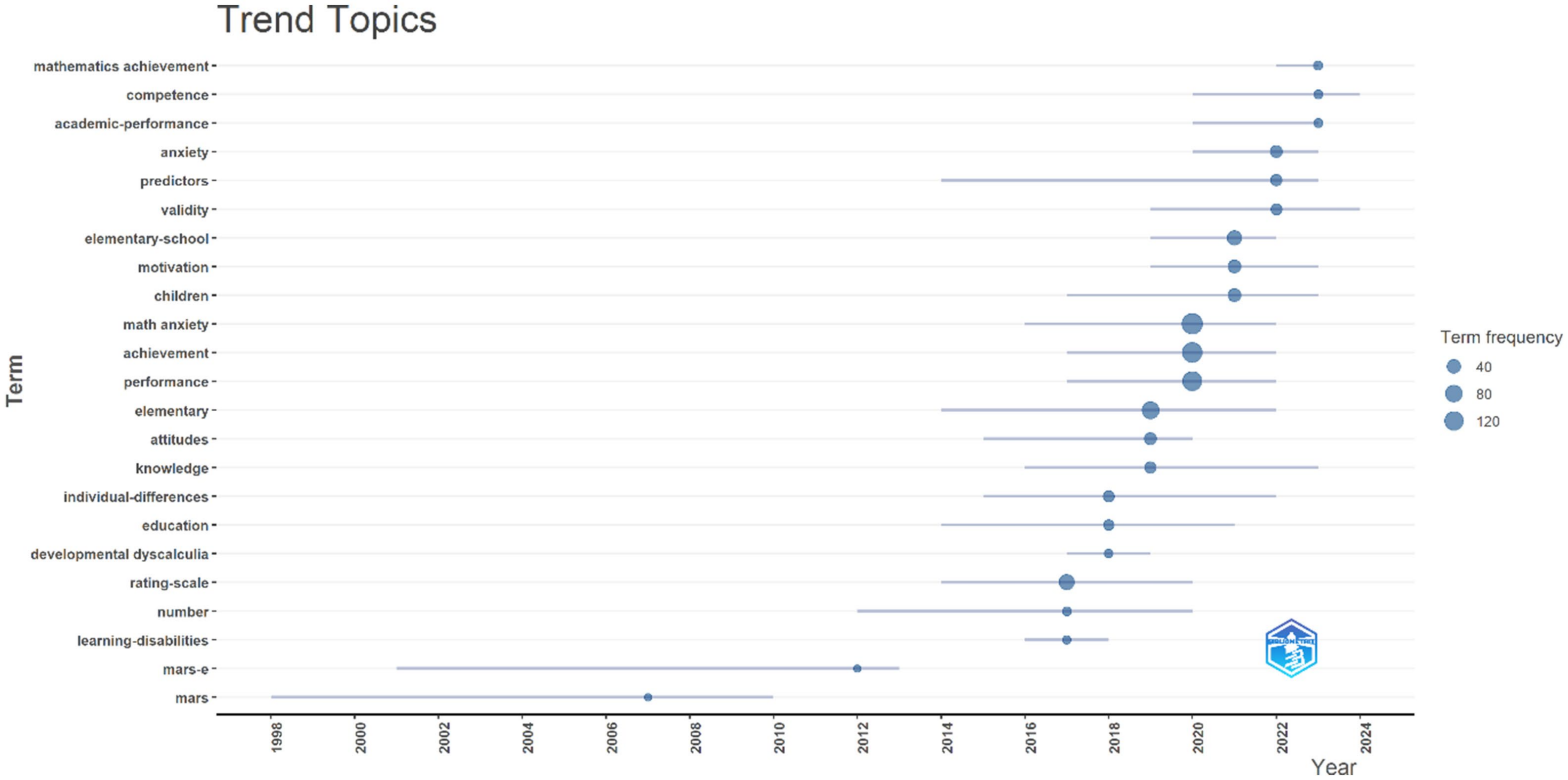
Keywords of trend topics on research field of mathematics anxiety in primary school.

In Figure 14, the trending keywords in the research on mathematics anxiety at the primary school level in the last decade are listed according to the years of publication.

The most frequently used keywords are ‘math anxiety’ (159), ‘achievement’ (138), ‘performance’ (123), ‘elementary’ (87), ‘elementary school’ (48), ‘children’ (33), ‘anxiety’ (27), ‘predictor’ (20), ‘validity’ (18), ‘individual differences” (18), ‘education” (14), ‘knowledge” (21), ‘competence” (7), ‘academic performance” (7), ‘mathematic achievement” (9), and ‘motivation” (35). The findings show that the keyword ‘math anxiety’ is used intensively, especially with terms such as ‘achievement,’ ‘performance,’ and ‘elementary.’ This result suggests that the context of students’ academic achievement, performance, and elementary school level are among the primary focus areas in mathematics anxiety research.

**Figure 15:**
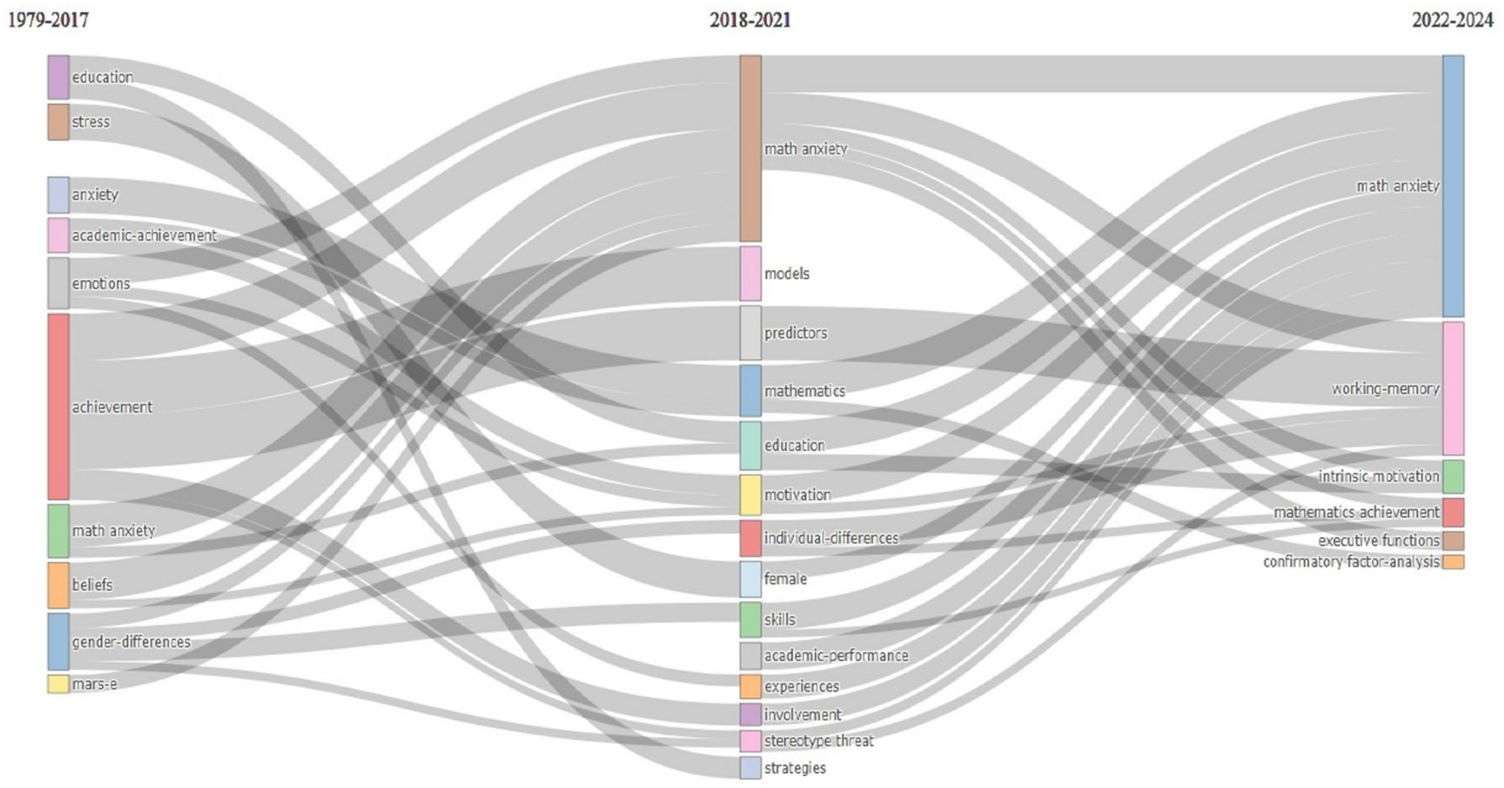
Thematic evolution of keywords in research field of mathematics anxiety in primary school.

The thematic evolution of the keywords in the research area of mathematics anxiety in primary school is presented in Figure 15. The research findings indicate that during the 1979–2017 period, academic achievement and the theme of’mathematics anxiety’ received significant attention. In the 2018–2021 period, it was determined that the research foci changed and the concepts of ‘gender stereotypes’ and ‘sex differences’ came to the fore, as well as themes such as ‘models’ and ‘predictors.’ The studies in this period addressed the relationship between mathematics anxiety and motivation in the context of gender roles and gender-based differences. Due to these themes, research from 1979 to 2017 focused a lot on how mathematics anxiety affected performance, while studies from 2018 to 2024 shifted towards more creative and psychological topics. In particular, cognitive processes such as “working memory,” “executive functions,” and “intrinsic motivation” were examined, along with their effects on students’ mathematical achievement and learning processes. These thematic shifts suggest that mathematics anxiety research has moved beyond a purely performance-oriented approach and has paid increasing attention to individual differences and cognitive processes.

## 4 Discussion

This study aims to examine the literature comprehensively on mathematics anxiety between 1979 and 2024, focusing on the primary school level, using bibliometric analysis methods. Since 1979, studies on mathematics anxiety have gained continuous momentum, and this area has established itself as a popular and researched thematic topic. The analysis of 404 studies from the Scopus and WOS databases showed the main research trends in mathematics anxiety, including key authors, important journals and institutions, and how researchers work together. The research helped us find the most important publications and authors in mathematics anxiety, which shows us what is popular in this area; looking at how countries and institutions work together reveals how studies on mathematics anxiety are spreading and where there are chances to collaborate internationally, and spotting popular topics in the literature gives us ideas for future research in this field.

It is seen that the interest in the field of research on mathematics anxiety in primary school has changed significantly over the years; this change has emerged as a continuous increase in the number of publications, and the literature in this field has been enriched with the collaboration of researchers from different disciplines.

According to the results of the research, the number of articles published varies according to years. The fact that a maximum of 7 articles were published annually between 1979 and 2009 shows that the subject attracted limited attention in this period and had not yet developed as a comprehensive research area. The steady increase between 2010 and 2016 suggests that the educational, psychological, and social effects of mathematics anxiety were considered more in this period and became a focus of interest among researchers. This rise can be linked to the growing importance of STEM (Science, Technology, Engineering, and Mathematics) in education, the understanding of how anxiety affects students’ math performance, and international tests (like PISA and TIMSS) highlighting math anxiety (Crato and Marôco, 2024). Since 2017, there has been a significant increase in the number of publications. This rapid increase indicates an intensifying academic interest in this field at the global level. The fact that the annual number of publications reached 34 articles in 2017 and increased to 96 before the end of 2024 indicates that the studies on mathematics anxiety have become multidimensional and turned into an interdisciplinary research area. Especially after the COVID-19 pandemic, the psychosocial conditions of students were examined more (Çaykuş and Çaykuş, 2020; Kımter, 2020; Şahin and Şahan, 2023), and research on the effect of mathematics anxiety on cognitive learning processes (Ashcraft et al., 2022; Pantoja et al., 2020) can be shown among the reasons for this increase.

In the word cloud that emerged as a result of the research, ‘math anxiety’ is one of the terms with the highest frequency. This finding shows that mathematics anxiety is at the center of the research in the field, and most of the studies on this subject are shaped around this main concept. The fact that the term ‘math anxiety’ stands out with 159 repetitions reveals that mathematics anxiety is a common problem, especially among primary school students, and the literature in this field focuses mainly on this issue (Commodari and La Rosa, 2021; Sagarduy et al., 2024; Tomasetto et al., 2021). The remarkable frequency of the terms ‘achievement’ and ‘performance’ in the word cloud indicates that mathematics anxiety has a significant effect on students’ academic achievement. There are also studies examining the relationship between mathematics anxiety and students’ mathematical achievement in the literature (Barroso et al., 2021; Israel and Olubunmi, 2014; Mutlu et al., 2017; Szczygieł and Pieronkiewicz, 2022; Türk and Bedir, 2021; Yenilmez and Özbey, 2006).

The thematic map analysis reveals important trends and research directions in mathematics education and related fields. The main themes in the first quadrant, such as ‘arithmetic children,’ ‘mathematical anxiety,’ ‘working memory,’ ‘math anxiety,’ ‘elementary school children,’ ‘mathematics,’ ‘mathematics anxiety,’ ‘primary school students,’ ‘emotions,’ and ‘mathematical achievement,’ reveal that these topics show both high development and centrality. These findings emphasize the importance of basic mathematical skills, especially for primary school students. This result clearly demonstrates the importance of educational programs for the development of basic mathematical skills at an early age. Studies indicate that the development of these skills can have positive effects on students’ long-term academic achievement by reducing their mathematics anxiety. Furthermore, further investigation of the relationship between working memory capacity and mathematical achievement may contribute to the development of educational strategies that are appropriate for students’ individual differences. The special themes in the second quadrant include ‘mathematics education,’ ‘preservice teachers,’ ‘elementary students,’ and ‘maths anxiety.’ This evidence shows that mathematics anxiety and preservice teachers’ preparation for mathematics education are still at the center of the field and that research in these areas constitutes an important focus for educational policies and teaching practices (Beisly et al., 2024; Brittain, 2021; Çelik, 2021; Özben and Kilicoglu, 2021; Yıldırım and Gürbüz, 2017). This indicates that teacher education programs should be supported with interventions aimed at reducing students’ mathematics anxiety. Moreover, the centrality of these themes suggests that educational policies should be restructured accordingly, taking into account the impact of mathematics anxiety on long-term academic and professional success. In the third quadrant, the themes of ‘elementary,’ ‘socioeconomic status,’ ‘teacher emotions,’ ‘number sense,’ ‘numerical cognition,’ ‘mathematic cognition,’ ‘teacher training,’ ‘academic mathematical attitudes,’ ‘science,’ ‘augmented reality,’ ‘learning environment,’ ‘STEM,’ and ‘longitudinal design’ are included. These results draw attention to the need for policy development to reduce the long-term effects of socioeconomic inequalities on mathematics education and the prominence of teachers’ views (Barroso et al., 2021; Coşkun and Özyer, 2023; Yüce et al., 2022). In addition, the inclusion of words related to developments in STEM and innovative approaches, such as augmented reality, indicates that modern tools that drive change in education have entered the research areas related to mathematics anxiety. How these tools can play a role in reducing mathematics anxiety and increasing students’ motivation to learn requires further research. The fourth quarter reveals a renewed focus on topics such as “rational numbers,” “primary education,” “distance learning,” and “self-regulated learning” have come to the fore again. These terms are characterized by their innovative features or their tendency to fade out of focus. However, these themes remain significant, reflecting a period of increasing investigation into distance education and student self-regulation skills in particular. These themes have become more prominent during the COVID-19 pandemic, when distance education has become widespread, so studies in these areas have contributed to shaping the new normal in education. Longitudinal studies can fill important gaps by examining in more detail how self-regulation skills affect mathematics achievement.

The journals contributing to the field of mathematics anxiety in primary school *are Frontiers in Psychology*, *Learning and Individual Differences*, *Contemporary Educational Psychology, Journal of Educational Psychology,* and *School Science and Mathematics*. It was determined that Frontiers in Psychology ranked first among the leading journals in the field of MA (Ersozlu and Karakus, 2019; Radević and Milovanović, 2024). This finding shows that Frontiers in Psychology aims to reach a wider readership by addressing a complex and multidimensional issue, such as mathematics anxiety in both psychological and educational contexts. At the same time, it can be said that this journal fills the gaps in the literature by hosting studies investigating the interdisciplinary effects of mathematics anxiety. In addition, the fact that this journal has published special issues on MA may have increased the number of publications in terms of quantity (Radević and Milovanović, 2024). Learning and Individual Differences (Radević and Milovanović, 2024), ranks second among journals that published special issues on MA with the number of publications on mathematics anxiety in primary school. In particular, the focus of this journal on individual differences provides an important reference point for designing personalized interventions for mathematics anxiety. This work once again highlights the need to develop effective strategies for students with different learning styles. The high number of publications in journals such as Contemporary Educational Psychology, Journal of Educational Psychology, and School Science and Mathematics shows that research in this area is not only addressed in the psychological dimension but also in the context of educational psychology and mathematics education (Christensen and Knezek, 2020; Li et al., 2021; Newcombe et al., 2009; Schukajlow et al., 2021). This diversity reveals that mathematics anxiety has been examined in a wide range from its effects on learning processes at the individual level to social and cultural dimensions. For example, these journals frequently address the role of restructuring teaching strategies or teacher training interventions in reducing this anxiety. In particular, investigating the effects of mathematics anxiety on learning processes and discussing how teaching strategies can be designed to reduce this anxiety are among the main topics addressed in the studies in these journals. In addition, it was determined that the most recent study on mathematics anxiety in primary school was published in the journal “Learning and Instruction.” This finding shows that current studies are still ongoing, as is and the dynamic structure of research in this field.

The analysis aims to reveal the geographical distribution of research productivity and the leading roles of universities contributing to publications in the field of MA in primary school. The results indicate that the University of Chicago, Beijing Normal University (BNU), and the University of Trieste are the leading contributing institutions in the field, respectively. This finding supports that the University of Chicago (USA), known for its pioneering studies on psychological and educational approaches in the field of mathematics anxiety, has made significant contributions to the literature (Ersozlu and Karakus, 2019; Sagarduy et al., 2024). This strong presence of the University of Chicago in research can be attributed to the institution’s development of innovative research methods by bringing together psychology and education disciplines. This university has pioneered projects that systematically examine the effects of mathematics anxiety on student performance and has brought a multidisciplinary perspective to research in this field. The fact that the most influential authors in the research findings and similar studies in the literature (Ersozlu and Karakus, 2019; Radević and Milovanović, 2024) are from the University of Chicago (USA) may explain the leading position of this university among the most contributing institutions. Moreover, the University of Chicago is home to prestigious centers and research groups in mathematics education. For example, one of the school’s renowned research centers, the *Center for Elementary Mathematics and Science Education (CEMSE)*, focuses on elementary mathematics education. This center has made a global contribution to the literature on mathematics anxiety through longitudinal studies in different age groups and international collaborations, resulting in a high volume of research on topics related to student development and psychology (Carey et al., 2019). BNU is one of the leading universities of education and psychology in China and has a large academic staff and research capacity in these fields (Gao, 2020). The fact that BNU ranks second in publications shows that research on mathematics anxiety has a strong foundation in Asia and that an international perspective has developed in this field. This strengthens China’s position in the global research ecosystem and provides important opportunities for understanding the effects of cultural contexts on mathematics anxiety. Research on China’s education system and student achievement provides a rich data set on how mathematics anxiety is addressed in cultural contexts. China is a country that places great importance on academic achievement (Liu et al., 2020). Mathematics, as one of the most focused subjects in this culture, can cause intense pressures on achievement (Boman, 2022). This reality makes research on issues such as math anxiety more critical. In this context, BNU has conducted numerous studies on mathematics anxiety to develop strategies to improve student achievement (Cheng et al., 2023; Qu et al., 2020). Given China’s population, researchers have easy access to large data sets. By collaborating with schools in the country, BNU can conduct comprehensive analyses on a large database, which may lead to more publications. The third place of the University of Trieste (Italy) in the ranking indicates the importance of research on mathematics anxiety in Europe (Caviola et al., 2022; Commodari and La Rosa, 2021; Hill et al., 2016). In Italy, there has been a long-standing debate about the need to reform mathematics education (Furinghetti and Menghini, 2023; Minisola, 2023). Within the scope of these reforms, research on students’ mathematics anxiety stands out as an important issue, and it is thought that it may have focused on primary school mathematics education and anxiety to meet the need. The University of Trieste’s contribution in this area is linked to the growing awareness in Europe of the importance of individual differences and emotional factors in mathematics education. This university has examined the impact of educational policies in studies on math anxiety and its consequences on student motivation (Cuder et al., 2024). In conclusion, these findings emphasize the importance of creating a research ecosystem in the field of mathematics anxiety that takes into account not only individual academic achievement but also countries’ educational policies and social cultures.

The results related to the cooperation network between institutions show that the University of Chicago is centrally located and carries out its studies in intensive cooperation with Stanford University, Columbia University, and Temple University. The intensity of the identified collaboration reveals that these universities work with an interdisciplinary approach (Diwakar and Babcock, 2021). This study shows that a complex phenomenon such as mathematics anxiety is too multidimensional to be handled within the boundaries of only one discipline. The importance of interdisciplinary collaboration becomes more evident with synergies in psychology, educational sciences, and neuroscience (Radević and Milovanović, 2024; Zuo et al., 2024). The network formed between these universities can facilitate filling knowledge gaps in the literature and developing new solutions. The collaboration between the University of Texas at Austin, Texas Tech University, and King’s College London provides an important example of how academic partnerships are shaped across regional boundaries (Wang et al., 2018). This collaboration emphasizes that mathematics anxiety is not only a local problem but should be addressed as a global educational and psychological challenge. Moreover, the inclusion of cultural, socio-economic, and educational factors from different regions in research can contribute to the development of universal and local solutions to mathematics anxiety. In the European context, the intensive collaboration between the University of Cambridge, the University of Trieste, and the University of Padua is noteworthy (Hill et al., 2016; Mammarella et al., 2019). This partnership demonstrates that research on mathematics anxiety in Europe allows for thorough studies that look at various aspects of mathematics anxiety, including thinking processes, education policies, and cultural factors (Ashcraft et al., 2022; Sagarduy et al., 2024). The strong European research network in this area also reveals that there are lessons to be learned from the education systems of different countries. The fact that the University of Cambridge has produced important studies on educational psychology issues such as mathematics anxiety (Carey et al., 2019; Hill et al., 2016; Mammarella et al., 2019), and that the Universities of Trieste and Padua have been involved in European Union projects to use technology in education, especially on pedagogical innovations in the field of STEM (science, technology, engineering, and mathematics), may have increased the cooperation between these universities (ATS STEM Project and EU STEM Coalition). Such collaborations foster theoretical knowledge and field strategies. For example, the development of innovative pedagogical methods to reduce math anxiety through STEM education has the potential to improve student achievement both in Europe and worldwide. Inter-university collaboration analyses reveal the multinational structure of MA research. These collaborations demonstrate that MA has universal characteristics as well as culture-specific dynamics (Hill et al., 2016; Radević and Milovanović, 2024). Furthermore, testing such international projects in different cultural contexts and sharing their results can contribute to the development of more inclusive and equitable policies in education.

The distribution of articles in the field of mathematics anxiety in primary schools by country reveals how the scientific production and influences in this field are shaped geographically. The USA is the most dominant country in this field with its high number of publications and the dominance of national-level studies, but it participates in international cooperation at a low rate. The USA is followed by China, Türkiye, Germany, Spain, and Germany (Linna et al., 2024; Yuan et al., 2023). While China produces a significant portion of its publications with international cooperation, it keeps the international share high despite having more national-level studies than the USA. Türkiye, on the other hand, produces most of its publications at the national level, indicating that the country focuses more on local studies and does not give enough space to international cooperation (Baş and Şahin, 2024; Uğraş, 2024). While Germany gives more importance to international collaboration (Frenzel et al., 2007), Spain encourages it more, despite its national-level studies (Brown et al., 2020; Sánchez-Pérez et al., 2021). These data reveal that the rates of national and international collaboration in the field of mathematics anxiety show significant differences between countries and that some countries are more oriented towards international research. The results generally indicate that the USA and European countries produce the majority of mathematics anxiety research. However, it is observed that the contributions of countries such as China and Türkiye are gradually increasing (Mildan and Aydoğdu, 2024; Uğraş, 2024; Zuo et al., 2024). In particular, the fact that Türkiye is a rising actor in this field reflects the increasing importance of the subject regionally and the effort to integrate more into the international literature (Altuntaş and Deringöl, 2021; Baş and Şahin, 2024; Çekmez et al., 2023; İlgün and Özcan, 2024; Mildan and Aydoğdu, 2024; Türk and Bedir, 2021). This trend suggests that academic policies and educational reforms in Türkiye are prioritizing important issues such as mathematics anxiety. It should also be emphasized that contributions from Asian and European countries can offer new perspectives in understanding the impact of different cultural and educational systems (Carey et al., 2016; Caviola et al., 2022; Cuder et al., 2024; Radević and Milovanović, 2024; Radišić et al., 2015).

Cross-country collaboration suggests that the USA is central to mathematics anxiety research, not only in terms of production but also in terms of its influence in the literature. European countries like the United Kingdom and Germany follow the USA (Dowker et al., 2016; Foley et al., 2017). Although Türkiye has relatively less influence on this list, it can be said to make a significant contribution in the regional context.

According to the results of the analysis, the article *“A Meta-Analysis of the Relationship between Anxiety towards Mathematics and Achievement in Mathematics’* by MA (1999) was the most cited and pioneering study in the field of mathematics anxiety in primary school. This research article filled an important gap in the literature by addressing the relationship between mathematics anxiety and mathematics achievement through meta-analysis. The number of citations it has received indicates that it has influenced subsequent research. This situation shows how much the research in the field needs methodologically systematic reviews and meta-analyses. The study titled *‘Female teachers’ math anxiety affects girls’ math achievement’* published by Beilock et al. (2010) ranks second with the number of citations. This article revealed the effects of female teachers‘math anxiety on girls’ math achievement and contributed to the field by reaching remarkable results in the fields of gender equality and teacher education. It can be said that this result draws attention to the fact that mathematics anxiety should be addressed not only in terms of students but also teacher-student dynamics in relation to teachers’ attitudes and behaviors. In particular, the fact that teachers’ anxiety indirectly affects student achievement points to the need to restructure teacher education programs. For instance, these studies recommend the development of specific intervention programs to assist pre-service teachers in managing mathematics anxiety. Furthermore, these results may provide a basis for interdisciplinary studies investigating the effects of gender roles on early education. Frenzel et al. (2007) published *“Girls and Mathematics - A “hopeless” issue? A control-value approach to gender differences in emotions towards mathematics’* ranked third with 350 citations. This article analyzes how emotions towards mathematics are shaped in the context of gender differences through control and value beliefs theory and examines gender differences in emotional context. The research shows that psychology and learning in this field have been evaluated remarkably. This study reveals not only the relationship between mathematics anxiety and gender but also the critical role of the meaning and value that an individual attributes to mathematics in academic achievement. In addition, the use of control-value theory can serve as a theoretical framework for designing educational policies. Overall, the results of the bibliometric analyses suggest that studies on mathematics anxiety at the primary school level often focus on contextual factors such as gender, teacher influence, and affective processes. However, it is suggested that future studies should address less examined issues such as how mathematics anxiety varies in different socio-cultural contexts and how this anxiety can be addressed, especially in students with low socio-economic status.

According to the research results, Beilock S., Levine S., Passolunghi M., Szucs D. and Ramirez G. are ranked as the most published authors in this field (Sagarduy et al., 2024). The authors’ work is geographically largely associated with prestigious institutions such as the University of Chicago (USA), University of Trieste (Italy), and University of Cambridge (UK). The evidence suggests that mathematics anxiety research is supported by academic collaborations in different countries (Carey et al., 2016; Caviola et al., 2022; Hill et al., 2016; Mammarella et al., 2019; Primi et al., 2020). The concentration of studies by authors with the highest number of publications is evident, particularly in the year 2018. This concentration in 2018 may be a reflection of the increased recognition of the effects of mathematics anxiety on educational policies and teaching strategies or the growing interest in student health in education and psychology. In terms of the number of citations, Beilock, S. and Ramirez, G. have authored influential and referential studies in this field. Levine, S. closely follows these two authors. These high citation rates reveal that these authors are important authorities in mathematics anxiety research and that their work has a wide academic impact. Beilock, S., in particular, has made significant contributions to the literature with his studies drawing attention to the effects of mathematics anxiety between teachers and students. This emphasizes that mathematics anxiety is not only an individual problem but also a multidimensional phenomenon that affects teachers‘pedagogical approaches and students’ academic achievement. As a result of the citation analyses of the countries, the USA ranked first, followed by the United Kingdom, Germany, and Canada (Lau et al., 2022). The USA and the UK consistently have the highest number of citations, ranking at the top among the most published institutions and authors. This finding suggests that universities in the USA and the UK have strong research infrastructures and that there is more funding and research support for issues such as math anxiety in these countries. For example, funding through the National Science Foundation (NSF) and similar organizations provides researchers with a wide range of opportunities and increases the research capacity of these countries (Dart-Kathios, 2024; Hildebrand and Cordes, 2024). The high performance of Germany, Canada, China, and Türkiye, while Italy is expected to be in the first rank of the countries with the highest number of citations, means that the institutions in Italy are less collaborative than the institutions and researchers in these countries. This result draws attention to the relationship between scientific productivity and collaboration. It can be stated that although Italy has high-quality individual studies, the impact of these studies is relatively limited due to the lack of international cooperation. It can be argued that collaborative efforts in countries such as Germany, Canada, China, and Türkiye have enabled these countries to make a wider contribution to the literature (Lau et al., 2022; Linna et al., 2024).

The collaborative network of the most prolific authors reveals the key figures driving the flow of knowledge. Authors such as Dowker, A., Beilock, S., Szucs, D., and Passolunghi, M. The fact that these authors collaborated with each other and with other academics and created a wide knowledge-sharing and discussion environment on the subject may have increased their effectiveness. According to the findings, authors such as Dowker, A., and Beilock, S. are in the most central position in the network. This indicates that they are important figures who direct the flow of information and establish critical links between other authors in the network. Such authors play a key role in providing resources for other researchers and initiating and guiding collaborations. For example, Beilock S’s central position provides important literature context for work on mathematics anxiety. The presence of stronger collaborations in some clusters and weaker collaborations in other clusters suggests that collaborations are not homogenously distributed. This suggests that some clusters in the network interact more than others and contribute to a larger pool of literature. Future research could develop strategies to encourage more collaboration opportunities with authors in isolated clusters.

According to the research results, the keywords “math anxiety,” “achievement,” “elementary” and “performance” came to the fore as trending topics. These words reflect an intense research interest in the effects of math anxiety on student achievement and performance. Mathematics anxiety is recognized as a psychological factor that can negatively affect students’ academic performance (Barroso et al., 2021; Cassady and Johnson, 2002; Commodari and La Rosa, 2021; Meinhardt and Pekrun, 2003; Papousek et al., 2012; Spielberger, 2019). These findings show that there is a strong trend in the literature regarding the relationship between mathematics anxiety and academic achievement and performance. This relationship in the literature emphasizes the role and impact of mathematics anxiety in learning processes. In particular, it has been frequently discussed that mathematics anxiety can negatively affect the way an individual approaches mathematical problems and the performance he/she exhibits in this process. The prominence of ‘achievement’ and ‘performance’ among the keywords shows that the studies on mathematics anxiety are largely related to these two factors. These studies have frequently addressed efforts to develop strategies to increase student achievement. However, some of these studies have directly linked the relationship between achievement and performance with the cognitive and emotional processes caused by mathematics anxiety. The findings that mathematics anxiety increases the cognitive load of the individual, which leads to a decrease in achievement and performance, are frequently found in the literature. Therefore, the fact that these three concepts are frequently used together in the literature shows that most of the research focuses on the effects of mathematics anxiety on student achievement and strategies for coping with it. In addition, the frequent use of keywords such as “elementary” and “elementary-school” indicates that the studies mostly focus on elementary school students and that this age group is considered a critical period in the context of mathematics anxiety. In addition, the use of keywords such as “predictor” and “individual differences” indicates that mathematics anxiety is associated with individual differences and their predictive effects on achievement. On the other hand, the fact that concepts such as “motivation” and “knowledge” have been among the trends in recent years reveals that mathematics anxiety is not only a performance-oriented problem but also has significant effects on students’ cognitive processes and motivation to learn. These findings suggest that research topics are increasingly being addressed in a broader psychological and cognitive context and that mathematics anxiety is considered a multidimensional phenomenon (Terry et al., 2023). In conclusion, these trend analyses reveal that research on mathematics anxiety focuses on the academic performance and individual differences of students at the primary school level, while factors such as motivation and knowledge acquisition are increasingly on the research agenda. These trends indicate that future research on mathematics anxiety can be addressed in a broader framework and with an interdisciplinary approach.

Research findings indicate that studies conducted in the field of mathematics anxiety at the elementary school level have undergone transformations over time, not only thematically but also theoretically and methodologically. The data obtained were examined by dividing them into three main periods. In the 1979–2017 period, most of the literature addressed mathematics anxiety (“mathematics anxiety”) in a performance-reduced plane, primarily in relation to the concepts of academic achievement (“achievement”) and performance. These methods were mainly based on traditional testing ideas, looking at how achievement, anxiety, and stress are connected, and they promoted simpler models that overlooked personal differences. This led to early studies in the field having limited impact in a pedagogical context. In other words, research on math anxiety during this period carried the traces of a research culture that focused solely on output and did not sufficiently understand learning processes.

The field faced structures involving more complex variables in the 2018–2021 period. The thematic focus during this period shifted to concepts such as “models,” “predictors,” “gender stereotypes,” and “sex differences.” Gender roles, students’ intrinsic motivation, self-efficacy perceptions, and academic self-concepts revealed that math anxiety is not only an individual challenge but also a socially constructed phenomenon. Research conducted during this period, particularly influenced by critical pedagogical approaches, has made it more apparent how students’ mathematical experiences are shaped within a social context. Therefore, these years have provided a new analytical ground for assessing math anxiety not only in terms of the individual’s cognitive capacity but also in terms of its interaction with social gender norms, expectations, and implicit messages of the education system.

The post-2022 period has witnessed a shift in mathematics anxiety research toward more innovative and multidimensional cognitive-psychological perspectives. Specifically, researchers are examining cognitive processes like working memory, executive functions, and intrinsic motivation to understand the underlying dynamics of mathematics anxiety. Studies include in-depth analyses that address math anxiety as a multidimensional cognitive-emotional experience. The way in which cognitive processes such as executive functions, working memory, self-regulation, and emotional awareness interact with math anxiety is leading to the development of more comprehensive and explanatory models in this field. This change has provided new theoretical insights into the causes of math anxiety and how it can be made functional or transformed. Research conducted within this framework has the potential to produce not only diagnostic but also transformative knowledge.

This thematic and theoretical evolution also demonstrates an increase in interdisciplinary collaboration in the field of math anxiety, with greater overlap between fields such as psychology, education, neuroscience, and gender studies. This highlights that educational research is becoming more inclusive and in-depth. For education systems, this transformation necessitates the development of more strategic policies based on understanding the multiple factors that influence students rather than merely focusing on measuring their anxiety levels.

In summary, the changes seen in the study of mathematics anxiety should be considered important not just for organizing existing research but also as a way to improve how we design research, train teachers, and develop educational programs. Future studies that take this multi-layered structure into account will contribute to the development of models that better understand students’ needs, flexibly adapt pedagogical practices to different variables, and aim to create environments that support mathematics learning.

## Data Availability

The raw data supporting the conclusions of this article will be made available by the authors, without undue reservation.
